# LRG1 Promotes Metastatic Dissemination of Melanoma through Regulating EGFR/STAT3 Signalling

**DOI:** 10.3390/cancers13133279

**Published:** 2021-06-30

**Authors:** Yuet Ping Kwan, Melissa Hui Yen Teo, Jonathan Chee Woei Lim, Michelle Siying Tan, Graciella Rosellinny, Walter Wahli, Xiaomeng Wang

**Affiliations:** 1 Centre for Vision Research, Duke NUS Medical School, 8 College Road, Singapore 169857, Singapore; joyce.kwan@duke-nus.edu.sg (Y.P.K.); melissateo@duke-nus.edu.sg (M.H.Y.T.); graciella.rosellinny@duke-nus.edu.sg (G.R.); 2 Singapore Eye Research Institute (SERI) The Academia, 20 College Road, Level 6 Discovery Tower, Singapore 169856, Singapore; 3 Pharmacotherapeutics Unit, Department of Medicine, Faculty of Medicine and Health Sciences, Universiti Putra Malaysia, Serdang 43400, Selangor, Malaysia; cheewoei@upm.edu.my; 4 Department of Surgery, Yong Yoo Lin School of Medicine, National University of Singapore, MD6, 14 Medical Drive, Singapore 117599, Singapore; surmts@nus.edu.sg; 5 Center for Integrative Genomics, Université de Lausanne, Le Génopode, CH-1015 Lausanne, Switzerland; walter.wahli@unil.ch; 6 Lee Kong Chian School of Medicine, Nanyang Technological University Singapore, Clinical Sciences Building, 11 Mandalay Road, Singapore 308232, Singapore; 7 Toxalim (Research Center in Food Toxicology), INRAE, ENVT, INP-PURPAN, UMR 1331, UPS, Université de Toulouse, F-31027 Toulouse, France; 8 Institute of Molecular and Cell Biology (IMCB), Agency for Science, Technology and Research (A*STAR), Proteos, 61 Biopolis Dr, Singapore 138673, Singapore

**Keywords:** leucine-rich α-2-glycoprotein-1, melanoma, metastasis, STAT3, EGFR

## Abstract

**Simple Summary:**

Melanoma is a highly metastatic and lethal form of skin cancer. Currently, there is no effective treatment available once melanoma cells spread to other parts of the body. Our study demonstrated that LRG1 regulates multiple aspects of melanoma metastasis through modulating EGFR/STAT3 signalling. Targeting LRG1 may offer an alternative way to control the metastatic spread of melanoma cells.

**Abstract:**

Although less common, melanoma is the deadliest form of skin cancer largely due to its highly metastatic nature. Currently, there are limited treatment options for metastatic melanoma and many of them could cause serious side effects. A better understanding of the molecular mechanisms underlying the complex disease pathophysiology of metastatic melanoma may lead to the identification of novel therapeutic targets and facilitate the development of targeted therapeutics. In this study, we investigated the role of leucine-rich α-2-glycoprotein 1 (LRG1) in melanoma development and progression. We first established the association between LRG1 and melanoma in both human patient biopsies and mouse melanoma cell lines and revealed a significant induction of LRG1 expression in metastatic melanoma cells. We then showed no change in tumour cell growth, proliferation, and angiogenesis in the absence of the host *Lrg1*. On the other hand, there was reduced melanoma cell metastasis to the lungs in *Lrg1*-deficient mice. This observation was supported by the promoting effect of LRG1 in melanoma cell migration, invasion, and adhesion. Mechanistically, LRG1 mediates melanoma cell invasiveness in an EGFR/STAT3-dependent manner. Taken together, our studies provided compelling evidence that LRG1 is required for melanoma metastasis but not growth. Targeting LRG1 may offer an alternative strategy to control malignant melanoma.

## 1. Introduction

Malignant melanoma is the most aggressive form of skin cancer [1,2] and the death rate of melanoma skin cancer patients is significantly higher than that of non-melanoma skin cancer patients [3]. Furthermore, the WHO predicts that death from melanoma will increase to 20% by 2025 [4]. Owing to the increase in life expectancy, ozone layer depletion, and low awareness of sun exposure, the number of death cases may escalate by 74% in 2040 [5]. Currently, there are only limited treatment options available for metastatic melanoma. Skin and gastrointestinal toxicity, as well as reduced efficacy due to resistance to immune and chemotherapies, are key challenges facing current melanoma drugs [6]. Therefore, there is an urgent need to identify novel therapeutic targets to improve the survival and quality of life of patients suffering from metastatic melanoma.

Our earlier study led to the identification of leucine-rich alpha 2 glycoprotein 1 (LRG1), a novel regulator of TGFβ1 signalling [7]. In endothelial cells (EC), LRG1 promotes angiogenesis by tipping the balance of TGFβ1 signalling toward the ALK1/Smad1,5,8 pathway, which is dependent on the presence of the type III TGF β1 receptor endoglin [7]. Besides its role in retinal angiogenesis [7], LRG1 has been linked to abnormal angiogenesis in glomerular [8], ischemic brain [9], cornea [10], and diabetic wounds [11]. Neovascularization plays an essential role in tumour expansion [12] and tumour vasculature provides a route of transportation for tumour cell dissemination [13]. Indeed, altered LRG1 expression is associated with various cancers and LRG1 has been proposed as a prognosis/diagnosis marker for hepatocellular carcinoma, gastric cancer, pancreatic cancer, leukaemia, ovarian cancer, breast cancer, prostate cancer, biliary tract cancer, bladder cancer, and non-small cell lung cancer [14,15,16,17,18,19,20,21]. LRG1 regulates tumour angiogenesis by inducing VEGFA through HIF1α activation [22]. Recently, angiogenesis-independent roles of LRG1 in tumourigenesis have also been reported. For instance, LRG1 regulates epithelial–mesenchymal transition (EMT) by activating RUNX1 [23] and TGFβ/Smad2,3 signalling [24,25,26]. However, the biological function of LRG1 in melanoma development and progression remains to be elucidated.

In the present study, we first investigated the expression of LRG1 in human melanoma biopsies and murine melanoma cell lines and established an association between LRG1 and melanoma. We then determined the role of LRG1 in melanoma growth in a tumour xenograft model as well as in vitro cell-based tumour cell viability and proliferation assays. Despite the important role of LRG1 in angiogenesis, there was no change in tumour angiogenesis and tumour growth in Lrg1-deficient mice. Instead, we found that LRG1 promotes melanoma cell migration, invasion, adhesion, and lung metastasis. Furthermore, both tumour cell and endothelial cell-derived LRG1 were important for tumour cell extravasation. Mechanistically, LRG1 exerts its function by regulating EGFR/STAT3 signalling, a central pathway involved in tumour metastasis. Stattic, a Stat3 inhibitor, completely abolishes the promoting effects of LRG1 on tumour cell activation. Targeting LRG1, therefore, may offer an alternative strategy to control STAT3-mediated melanoma metastasis.

## 2. Materials and Methods

### 2.1. Mice

*Lrg1^-^*^/*-*^ mice used in this study were originally generated by the University of California Davies Knockout Mouse Project (KOMP) repository (https://www.mmrrc.org/catalog/sds.php?mmrrc_id=48463) and were a generous gift from Professors John Greenwood and Steven Moss at UCL Institute of Ophthalmology. Animal care and procedures were performed under the guidelines of the Institutional Animal Care and Use Committee (IACUC, Protocol number: A0269) of the Nanyang Technological University in Singapore and the Guide for Care and Use of Laboratory Animals from the US National Institutes of Health. All mice were housed in an environmentally controlled room (22 °C, 40–60% humidity, and a 12-h light cycle).

### 2.2. Tissue Microarray and Immunohistochemistry

Human skin cancer and normal tissue arrays (cat#SK721) were purchased from US Biomax (Rockville, MD, USA). The paraffin-embedded slides were deparaffinized and rehydrated before being subjected to antigen retrieval in a 10 mM citrate buffer (pH 9.0) under boiling conditions for 25 min. The slides were then incubated with 3% hydrogen peroxide (Sigma Aldrich, Burlington, MA, USA) for 10 min followed by blocking with 10% blocking buffer containing horse serum for 30 min before being incubated with anti-LRG1 antibodies (1:100, Proteintech, Rosemont, IL, USA) overnight at room temperature. The next day, the unbound primary antibodies were washed off and the section was incubated with HRP-conjugated secondary antibodies followed by treatment with a substrate reagent containing diaminobenzidine (DAB) for 5 min (Dako Real Envision Detection Kit). The section was counterstained with hematoxylin, dehydrated, and mounted with Leica Ultra CV mounting media (Leica, Wetzlar, Germany).

### 2.3. Cell Lines and Cell Culture Conditions

Mouse melanoma cell lines B16F0 and B16F10 and the human melanoma cell line A375 were obtained from the American Type Culture Collection (Manassas, VA, USA). Cells were cultured in Dulbecco’s modified Eagle’s medium (DMEM; Gibco, USA) supplemented with 10% fetal bovine serum (FBS; Gibco, USA), 2 mM of l-glutamine (Gibco, USA), 100 U/mL of penicillin, and 100 µg/mL of streptomycin (Nacalai Tesque, Kyoto, Japan). Human pulmonary microvascular endothelial cells (HPMEC) were obtained from Promocell (Heidelberg, Germany) and cultured in Endothelial Cell Medium-2 supplemented with endothelial cell growth medium bullet kits (Lonza, Basel, Switzerland). All cell lines were maintained at 37 °C in a humidified atmosphere of 95% air and 5% CO_2_.

### 2.4. Chemicals

The chemicals used were erlotinib (Sigma-Aldrich), FAK inhibitor 14 (Sigma-Aldrich), Src-I1 (Sigma-Aldrich), and stattic (Sigma-Aldrich). Erlotinib, Src-I1, and stattic were dissolved in dimethylsulfoxide (DMSO), while FAK inhibitor 14 was dissolved in water at the desired concentrations and stored at −20 °C.

### 2.5. Molecular Biology Methods

The coding sequence of human LRG1 (NM_052972) carrying a 6xHis-tag at the 3′ end and a Kozak consensus sequence at the 5′ end was cloned into pcDNA3.1 (Invitrogen, Waltham, MA, USA) at the HindIII/XhoI sites to form pcDNA-hLRG1. The coding sequence of mouse Lrg1 (NM_029796) carrying a 6xHis-tag at the 3′ end and a Kozak consensus sequence at the 5′ end was cloned into pcDNA3.1 (Invitrogen, Waltham, MA, SA) at the HindIII/XbaI sites to form pcDNA-mLrg1. Cells were transfected with pcDNA-hLRG1 or pcDNA-mLrg1 plasmid (2500 ng) using Lipofectamine 2000 (Invitrogen, Waltham, MA, USA) according to the manufacturer’s protocol. Small interfering RNA against Lrg1 (siLrg1; L-015179-01-0010; ON-TARGETplus SMARTpool human LRG1 siRNA) and non-targeting siRNA (negative control, siScr: D-001810-01-20; ON-TARGETplus Nontargeting siRNA#1) were purchased from Dharmacon (Lafayette, LA, USA). Cells were transfected with the siRNA oligonucleotides (25 nM) using Lipofectamine RNAiMAX transfection reagent (Life Technologies, Carlsbad, CA, USA), based on the manufacturer’s protocol.

### 2.6. Cell Viability Assay

The CellTiter 96^®^ AQueous One Solution Cell Proliferation Assay (MTS) (Promega, Madison, WI, USA) was used according to the manufacturer’s instructions. In brief, cells were seeded at 5 × 10^3^ cells/well in a 96-well flat-bottom plate. The next day, cells were starved for 6 h to synchronize cell growth before being cultured in full growth media. At each time point, 20 uL of MTS reagent was added into each well and the plate was incubated at 37 °C for 2 h in a humified, 5% CO_2_ atmosphere. The absorbance at 490 nm was recorded using a microplate reader (Tecan, Mannedorf, Switzerland).

### 2.7. Cell Proliferation Assay

Cells were seeded at a density of 4.5 × 10^3^ cells/well in a 48-well plate. The next day, cells were serum-starved in DMEM supplemented with 0.5% FBS for 6 h before being cultured in full growth media for another 72 h. Cells were then washed with PBS and fixed with 4% paraformaldehyde for 20 min at room temperature before being washed again in PBS and blocked with staining buffer containing 1% BSA, 1% Tween 20, and 3% Triton-X in PBS. One hour later, cells were incubated with Ki67 antibodies (Abcam, UK) at 1:500 dilution overnight. Cells were washed with staining buffer before being incubated with Goat anti-Rabbit IgG (H+L) Cross-Adsorbed Secondary Antibody, Alexa Fluor 594 (Thermo Fischer Scientific, Waltham, MA, USA, cat#A11012) at 1:500 dilution and 4,6-diamidino-2-phenylindole (DAPI). Five random fields of 10× objective images were taken using a Nikon Ti-E fluorescence microscope (Nikon, Tokyo, Japan). Cell proliferation rate was calculated as the percentage of Ki67 positive cells of the total cell number per well as determined using the cell counter plugin of the Image J software (National Institutes of Health, Bethesda, MD, USA).

### 2.8. Cell Migration and Invasion Assays

Cell migration and invasion assays were performed using 24-well plates with 8 μm pore size transwell inserts (Corning Life Science, Corning, NY, USA). For migration assays, 8 × 10^4^ cells in serum-free DMEM medium were seeded into the upper chamber of the transwell, while NIH-3T3 cell-conditioned medium was applied to the lower chamber of the transwell as a chemoattractant. For invasion assays, the inserts were coated with 1:10 diluted Matrigel (50 μL/well) (BD Biosciences, Franklin Lakes, NJ, USA) and kept at 37 °C for 2 h to allow polymerisation to occur before seeding cells into the transwell. Cells were fixed in 1% paraformaldehyde (PFA), permeabilised in 0.5% Triton X after 4 h for migration assays or 18 h for invasion assays. Non-migrating or invading cells on the top of the transwell membrane were gently removed using cotton swabs while the migrated/invaded cells at the bottom of the membrane were stained with DAPI. Migrated/invaded cells were visualised under a Nikon Ti-E fluorescence microscope (Nikon, Tokyo, Japan) Five fields from each insert were captured and quantified using the Image J software.

### 2.9. Transendothelial Migration Assay

One × 10^5^ HPMEC cells were plated in the upper chamber of a collagen-coated transwell insert with 8 µm pore size (Corning Costar, Cambridge, MA, USA) and grown in the complete endothelial medium for 4 days to reach 100% confluence. Melanoma cells were pulsed with 25 µM CellTracker Green CMFDA (Invitrogen, Waltham, MA, USA) dye for 30 min before being trypsinised and plated on top of the HPMEC monolayer. Cells were allowed to migrate for 24 h toward the complete DMEM in the lower chamber of the transwell. Transwell inserts were fixed with 1% PFA and permeabilised by 0.5% Triton X-100. Non-migrated cancer cells were removed from the upper side of the filter using cotton buds. Migrated cells on the lower side of the filter were visualised under a Nikon Ti-E fluorescence microscope (Nikon, Tokyo, Japan). Five fields from each insert were captured and quantified using the Image J software.

### 2.10. Xenograft Tumour Model

B16F10 cells at a density of 2 × 10^6^ cells were injected subcutaneously into the left flank of six- to eight-week-old wild-type and *Lrg1^-^*^/*-*^ mice. Mice were monitored daily. Once tumours became visible, they were measured using a caliper for two weeks. Mice were sacrificed once the tumour size reached 250 mm^3^.

### 2.11. Lung Metastasis Model

Five × 10^5^ of B16F10 cells in 200 µL of PBS were intravenously inoculated into 6- to 8-week-old wild-type and *Lrg1^-^*^/*-*^ mice. The mice were sacrificed two weeks after the inoculations.

### 2.12. Extravasation Assay

One × 10^6^ B16F10 cells labelled with CellTracker Green CMFDA (Invitrogen, Waltham, MA, USA) were intravenously inoculated into 6- to 8-week-old wild-type and *Lrg1^-^*^/*-*^ mice. Mice were sacrificed and their lungs were harvested 24 h post-inoculation. Lung tissues were histologically processed and the number of labelled B16F10 cells were determined using a Nikon Ti-E fluorescence microscope (Nikon, Tokyo, Japan).

### 2.13. Western Blot

Cells were lysed in RIPA buffer containing protease inhibitor cocktail-EDTA free (Nacalai Tesque, Kyoto, Japan, cat#03969-21) and phosphotase inhibitor cocktail-EDTA free (Nacalai Tesque, Kyoto, Japan, Cat#07575-51). Protein concentration was determined via the Bradford method. Eighty micrograms of protein was separated via SDS-PAGE and transferred to PVDF membranes (Milipore, Burlington, MA, USA). The membranes were then incubated with p-STAT3 (Tyr705) antibodies (Cell Signaling Technology, Danvers, MA, USA, cat#9145), STAT3 antibodies (Cell Signaling Technology, Danvers, MA, USA, cat#30835), phospho-Src (Tyr527) antibodies (Cell Signaling Technology, Danvers, MA, USA, cat# 2105), Src antibodies (Cell Signaling Technology, Danvers, MA, USA, cat#2108), and GAPDH antibodies (Santa Cruz Biotechnology, Santa Cruz, CA, USA, cat#32233) at 4 °C overnight followed by horseradish peroxidase (HRP)-labelled secondary antibodies (Santa Cruz Biotechnology, Santa Cruz, CA, USA) at room temperature for 2 h.

### 2.14. Quantitative Real-Time PCR (qRT-PCR)

Total RNA was extracted using RNAzol RT (Molecular Research Center, Albany, NY, USA). RNA concentrations were determined using a Nanodrop 2000C Spectrophotometer 19 (Thermo Fisher Scientific, Waltham, MA, USA). The cDNA was synthesized using qScript cDNA Supermix (Quantabio, Beverly, MA, USA) according to manufacturer protocols. The qRT-PCR was conducted with SYBR Green (PrimerDesign Precision, Chandler’s Ford, UK) using a QuantStudio 6 Flex Real-Time PCR System (Life Technologies, Carlsbad, CA, USA). Data were analysed using the 2 (-Delta Delta C(T)) method. The primers used in this study are listed in Table 1.

### 2.15. Histology and Immunofluorescence Staining

Resected tumours and lungs were fixed in 4% PFA for 24 h, washed with PBS, and gradually transferred to 15% sucrose followed by 30% sucrose before being embedded in O.C.T. compound (Thermo Fisher Scientific, Waltham, MA, USA). Six-micrometre cryosections of tumour samples were dehydrated and blocked with a blocking buffer containing 2% BSA, 1% Tween 20, 3% Triton X, and horse serum for an hour before being incubated with primary antibodies followed by a washing step and then incubated with secondary antibodies. The primary antibodies used were Ki67 (Abcam, Cambridge, UK, cat#ab15580) and CD31 (BD Biosciences, Franklin Lakes, NJ, USA, cat#553370). The secondary antibodies employed were Goat anti-Rabbit IgG (H+L) Cross-Adsorbed Secondary Antibody, Alexa Fluor 594 (Thermo Fischer Scientific, Waltham, MA, USA, cat#A11012), and Goat anti-Rat IgG (H+L) Cross-Adsorbed Secondary Antibody, Alexa Fluor 488 (Thermo Fischer Scientific, Waltham, MA, USA, cat#A11006). The nuclei were stained with DAPI. The slides were subsequently washed with PBS, mounted with Mowiol, and visualised under a Zeiss LSM 800 inverted confocal microscope or a Nikon Ti-E fluorescence microscope (Nikon, Tokyo, Japan). Images were captured at 20× magnification. Haematoxylin-eosin staining was performed to quantify the total area and the number of metastases in each lung using the ImageJ software (National Institutes of Health, Bethesda, MD, USA).

### 2.16. Statistical Analyses

Data are represented as mean ± standard error of mean (SEM). Statistical comparison of results was performed via two-tailed, unpaired Student’s *t*-test using Prism 8.0 (GraphPad, La Jolla, CA, USA). Statistical significance is denoted with asterisks as follows: * *p* ≤ 0.05; ** *p* ≤ 0.01; *** *p* ≤ 0.001 and **** *p* < 0.0001.

## 3. Results

### 3.1. LRG1 Levels Are Highly Enhanced in Melanoma Cells

**Figure 1:**
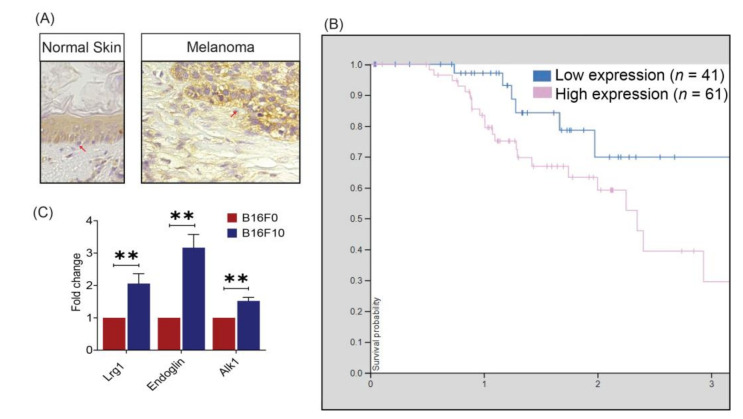
LRG1 expression in human melanoma biopsies and mouse melanoma cell lines. (**A**) Representative photomicrographs of immunohistochemistry staining demonstrating the expression pattern of LRG1 in normal human skin and malignant melanoma tissue. (**B**) Kaplan–Meier curves showing stratification of three-year survival probability as a function of *LRG1* RNA expression (adapted from The Human Protein Atlas). (**C**) Real-time quantitative PCR analysis of *Lrg1, endoglin*, and *Alk 1* expression. Data are presented as the mean ± S.E.M of three independent experiments. Statistical analysis was performed via two-tailed, unpaired Student’s *t*-test; ** *p* < 0.01.

To establish the association between LRG1 and melanoma, we investigated the expression of LRG1 in human biopsy specimens via immunohistochemistry staining using an LRG1-specific antibody. First, our study showed that LRG1 is expressed in melanocytes of normal human skin tissues but at a slightly lower level compared to neighbouring keratinocytes (Figure 1A). Second, LRG1 is much enhanced in melanoma cells of malignant melanoma tissue as compared to cells in the stroma (Figure 1A). Consistent with this observation, data from The Human Protein Atlas (https://www.proteinatlas.org/, accessed on 23 April 2021) show that a higher expression of *LRG1* is associated with poor three-year clinical outcomes in patients with melanoma (Figure 1B). We further tested Lrg1 expression in two different murine melanoma cell lines, B16F0 and B16F10. B16F10 cells are more aggressive and highly metastatic compared to B16F0 cells [27,28,29,30]. As demonstrated via qRT-PCR, higher *Lrg1* mRNA levels are observed in B16F10 cells compared to B16F0 cells (Figure 1C). Our previous study showed that LRG1 exerts its function in endothelial cells (EC) through interaction with the TGFβ type III receptor, Endoglin [7]. Similar to *Lrg1*, *Endoglin* is highly expressed in metastatic B16F10 cells (Figure 1C). Interestingly, the expression of TGFβ type I receptor *Alk1* is also significantly higher in B16F10 cells (Figure 1C). It is worth noting that the expression levels of TGFβ type I receptor *Alk5* is comparable in B16F10 and B16F0 cells (Supplementary Figure S1).

### 3.2. Host Lrg1 Deficiency Has No Impact on Melanoma Growth

**Figure 2:**
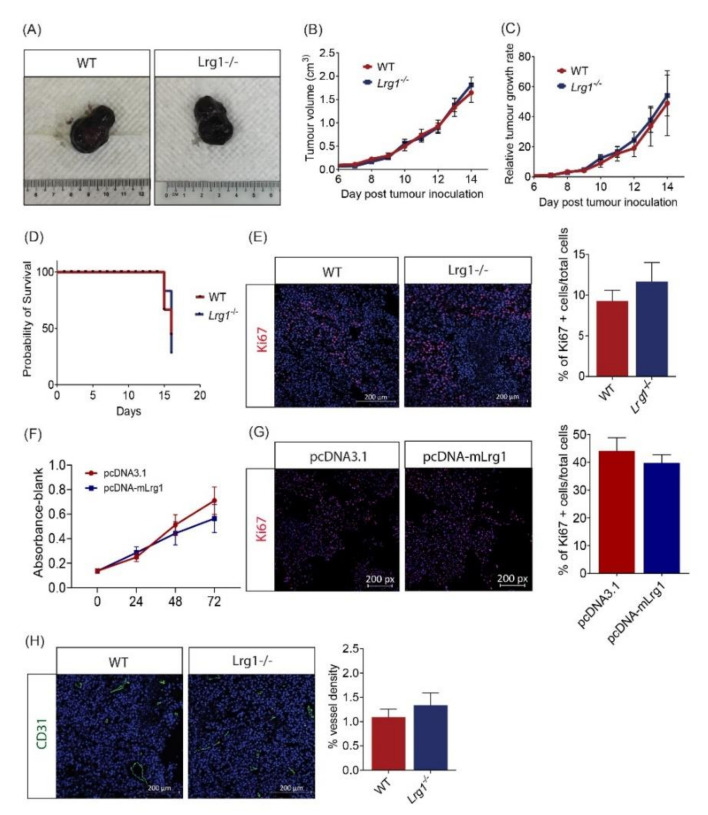
Host *Lrg1*-deficiency does not affect tumour growth in a tumour xenograft model. (**A**) Representative images of B16F10 tumours dissected from wild-type and *Lrg1^-^*^/*-*^ mice. (**B**) Tumour volume was plotted over time in both experimental groups. (**C**) Tumour growth rates were determined based on changes in tumour volume over time in both experimental groups. (**D**) Kaplan–Meier survival curves of wild-type or *Lrg1^-^*^/*-*^ tumour-bearing mice. (**E**) Representative images (left) and quantification (right) of the percentage of Ki67+ cells (red) in tumour samples collected from wild-type and *Lrg1^-^*^/*-*^ mice. Nuclei were labelled with DAPI (blue). (**F**) MTS assay was performed on B16F10 cells transfected with pcDNA-mLrg1 or pcDNA3.1 control plasmid at 24, 48, and 72 h post-transfection. (**G**) Representative images (left) and quantification (right) of Ki67+ cells (red) in Lrg1 overexpressing or pcDNA3.1 plasmid transfected control B16F10 cells. (**H**) Representative images (left) and quantification (right) of CD31+ vessel (Green) in tumour samples collected from wild-type or *Lrg1^-^*^/*-*^ mice. All images are representative. Data are presented as the mean ± SEM. of three independent experiments. Statistical analysis was performed via two-tailed, unpaired Student’s *t*-test.

Murine melanoma cell line B16F10 is highly malignant and has been widely used to study melanoma growth and metastasis. In this project, B16F10 cells were inoculated subcutaneously into the flank of wild-type and *Lrg1*^−/−^ mice. Once tumours became visible, tumour volume was measured daily for a continuous 14 days using a caliper. Surprisingly, no significant differences in tumour volume (Figure 2A,B) and growth rate (Figure 2C) were observed between wild-type and *Lrg1*^−/−^ mice. Additionally, the Kaplan–Meier survival curves did not show any difference between mice from the two experimental groups (Figure 2D). This was further confirmed by immunofluorescence staining with the cell proliferation marker Ki67. The percentage of Ki67+ cells in tumour tissues collected from *Lrg1*-deficient mice was comparable to that in wild-type controls (Figure 2E). To complement these in vivo observations, the role of LRG1 in tumour cell proliferation was investigated in vitro. The MTS assay was used to evaluate the viability of B16F10 cells transfected with pCDNA3.1-mLrg1, which resulted in Lrg1 overexpression, or pcDNA3.1 control plasmid; no difference was observed between the two experimental groups (Figure 2F). Furthermore, plasmid-mediated Lrg1 overexpression did not affect B16F10 cell proliferation as demonstrated by the percentage of Ki67+ cells (Figure 2G). Similar observations were made in the human melanoma cell line A375 (Supplementary Figure S2A,B).

Tumours require access to blood vessels to grow beyond 2 mm^3^ [31]. Considering the role of LRG1 in ocular angiogenesis [7], we next investigated whether tumour vascularization is affected in wild-type and *Lrg1^-^*^/*-*^ mice. To our surprise, there was no change in tumour vessel density in the absence of host Lrg1 compared to wild-type mice as demonstrated by similar CD31 positive areas (Figure 2H). Together, these data suggest that host Lrg1-deficiency does not affect tumour cell viability, proliferation, and tumour angiogenesis.

### 3.3. Host Lrg1 Deficiency Leads to Reduced Pulmonary Metastasis of Melanoma In Vivo

**Figure 3:**
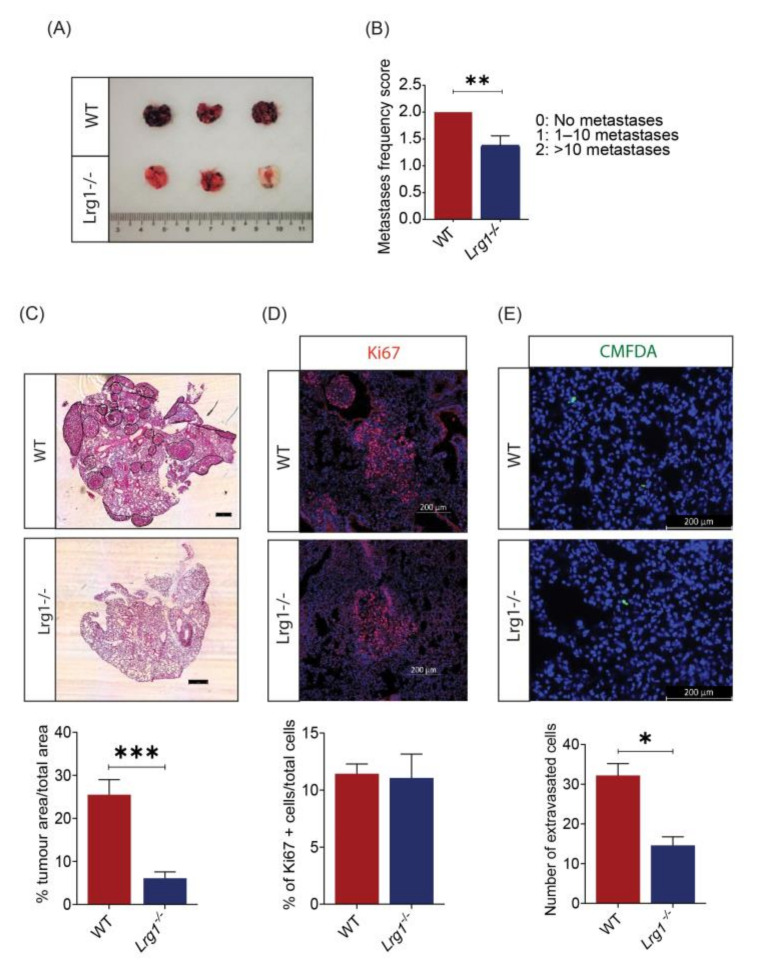
Host *Lrg1* deficiency reduces pulmonary metastasis of melanoma in vivo. (**A**) Representative images of lung metastases in wide-type and *Lrg1^-^*^/*-*^ mice. Nodules are easily identifiable due to their black pigmentation. (**B**) Quantification of lung metastases frequency in wide-type and *Lrg1^-^*^/*-*^ mice. (**C**) Representative images of haematoxylin-eosin staining (top) and quantification (below) of the metastatic burden of lung tissues in wide-type and *Lrg1^-^*^/*-*^ mice. The percentage of tumour nodule area over the total lung area was measured. (**D**) Representative images (top) and quantification (below) of Ki67+ cells (red) in lung tissues of wide-type and *Lrg1^-^*^/*-*^ mice. (**E**) Representative images of lung sections (top) to visualise extravasated melanoma cells identified via CMFDA Green signal (green) and quantification (below) of the number of extravasated cells out from blood vessel 24 h following intravenous injection of B16F10. Nuclei were labelled with DAPI (blue). Data are presented as the mean ± SEM. Statistical analysis was performed via two-tailed, unpaired Student’s *t*-test; * *p* < 0.05, ** *p* < 0.01; *** *p* < 0.001. Wildtype group: *n* = 9, *Lrg1^-^*^/*-*^ group: *n* = 8.

As melanoma is a highly metastatic disease, we then explored the potential role of Lrg1 in melanoma metastasis. Lung metastasis was induced in wild-type and *Lrg1^-^*^/*-*^ mice via intravenous delivery of B16F10 cells. Pulmonary metastases can be visualised under the dissection microscope. There is a clear difference in the number of metastatic nodules with black pigmentation between wild-type and *Lrg1^-^*^/*-*^ mice (Figure 3A). The number of pulmonary metastatic nodules was then counted and represented as metastasis frequency following a scoring system by denoting 0 for no metastases, 1 for within 10 metastases, and 2 for more than 10 metastases. Our study revealed a significantly lower metastasis frequency score in *Lrg1^-^*^/*-*^ mice as compared to that in wild-type mice (Figure 3B). Concomitant with this observation, haematoxylin-eosin staining of excised lungs showed a similar reduction in the percentage of tumour nodule area in the lungs of *Lrg1^-^*^/*-*^ mice following the histopathological analysis (Figure 3C). Further analysis of the percentage of Ki67+ cells in the lung samples of wild-type and *Lrg1^-^*^/*-*^ mice showed no significant changes, suggesting that post-extravasation proliferation was not affected (Figure 3D). Finally, the extravasation capability of melanoma cells was studied by inoculating CMFDA Green-labelled B16F10 cells through the tail vein of wild-type and *Lrg1^-^*^/*-*^ mice. The number of extravasated B16F10 cells was visualised under epifluorescence microscopy 24 h post-inoculation and quantified. There was a significant reduction in the number of extravasated B16F10 cells in *Lrg1^-^*^/*-*^ mice compared to that in wild-type mice (Figure 3E). Taken together, these data provided compiling evidence that Lrg1 is required for melanoma metastasis into the lungs in vivo.

### 3.4. Lrg1 Promotes B16F10 Cell Invasiveness In Vitro

**Figure 4:**
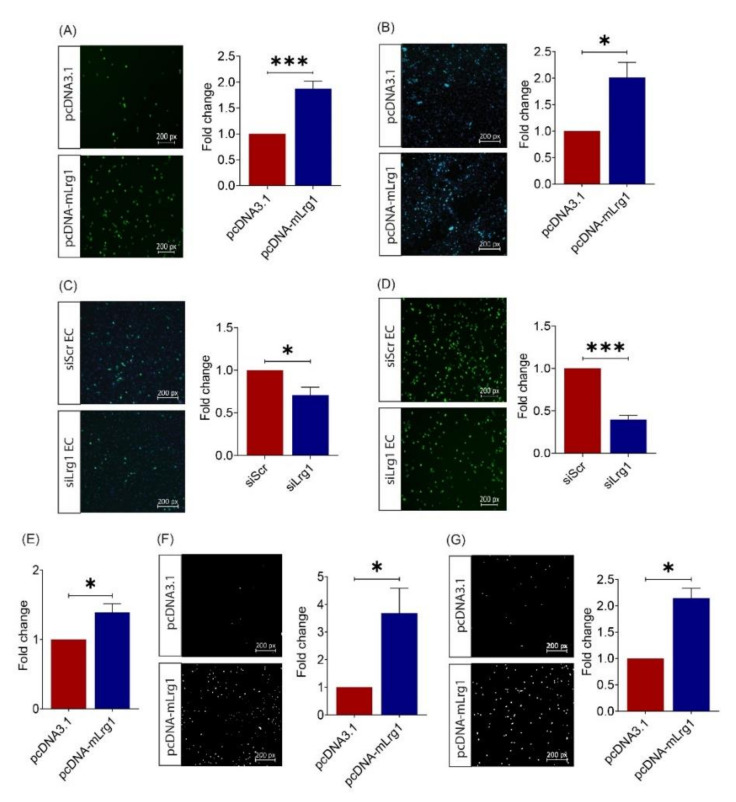
Lrg1 promotes B16F10 cell invasiveness in vitro. (**A**) Representative images and quantitative analysis of the number of CMFDA-positive Lrg1 overexpressing or control B16F10 cells adhered to the HPMEC monolayer. (**B**) Representative images and quantitative analysis of the number of CMFDA-positive Lrg1 overexpressing B16F10 cells that migrated through the HPMEC monolayer. (**C**) Representative images and quantitative analysis of the number of CMFDA-positive B16F10 cells adhered to the HPMECs subjected to siRNA-mediated LRG1 knockdown. (**D**) Representative images and quantitative analysis of the number of CMFDA-positive B16F10 cells that migrated through the HPMECs subjected to siRNA-mediated LRG1 knockdown. (**E**) Quantitative analysis of Lrg1 overexpressing or control B16F10 cells adhered to fibronectin. (**F**) Representative images and quantitative analysis of invaded Lrg1 overexpressing and control B16F10 cells using the Matrigel invasion assay. (**G**) Representative images and quantitative analysis of migrated Lrg1 overexpressing or control B16F10 cells in Transwell. Migrated and invaded cells are labelled by DAPI. All images are representative. Data are presented as mean ± SEM. of three independent experiments. Statistical analyses were performed via two-tailed, unpaired Student’s t-test. * *p* < 0.05; *** *p* < 0.001.

Having established a role for Lrg1 in melanoma metastasis, especially the extravasation step, in vivo, we next investigated how Lrg1 modulates melanoma cell function in vitro. The ability of circulating tumour cells to tether to the vasculature is a prerequisite step for the extravasation [32]. Consistent with the increased invasiveness, there was a significantly higher number of Lrg1 overexpressing B16F10 cells that adhered to the human pulmonary microvascular endothelial cell monolayer in the in vitro assay (Figure 4A). The adhered tumour cells would then migrate through the endothelium to eventually settle at secondary sites. Our study showed that Lrg1 overexpressing B16F10 cells were more prone to transmigrate through the HPMEC monolayer (Figure 4B). To mimic the previous observations in vivo, HPMECs were subjected to siRNA-mediated LRG1 knockdown (Supplementary Figure S3). Our study showed that the ability of B16F10 cells to adhere to (Figure 4C) or migrate (Figure 4D) through the siLrg1-treated HPMEC monolayer was significantly compromised.

The success of colonization at the secondary tumour site following extravasation depends on the ability of tumour cells to adhere to the extracellular matrix (ECM), degrade ECM components, and move around [33]. This prompted us to test the impact of Lrg1 on melanoma cell adhesion to ECM. Fibronectin is a major component of the tumour ECM and plays key regulatory roles in the tumour matrisome [34]. As shown in Figure 4E, there was an increased number of Lrg1 overexpressing B16F10 cells that adhered to fibronectin. Next, we performed the Matrigel invasion assay to test the role of Lrg1 in melanoma cell invasion. Consistent with the results presented above, Lrg1 overexpression significantly increased the invasiveness of B16F10 cells (Figure 4F). Furthermore, a transwell migration assay was performed using pcDNA3.1-mLrg1 or pcDNA3.1 control plasmid transfected B16F10 cells. As compared to the pcDNA3.1 transfected controls, Lrg1 overexpressing B16F10 cells showed increased motility (Figure 4G). We further confirmed LRG1’s role in tumour cell adhesion to the endothelium (Supplementary Figure S2C), transendothelial migration (Supplementary Figure S2D), invasion (Supplementary Figure S2E), and migration (Supplementary Figure S2F) in a human melanoma cell line A375. Together, these data demonstrated that Lrg1 promotes tumour cell dissemination by affecting various properties of melanoma cells in both mice and humans.

### 3.5. Lrg1-Induced Activation of the EGFR/STAT3 Pathway Is Required for Melanoma Cell Invasiveness

**Figure 5:**
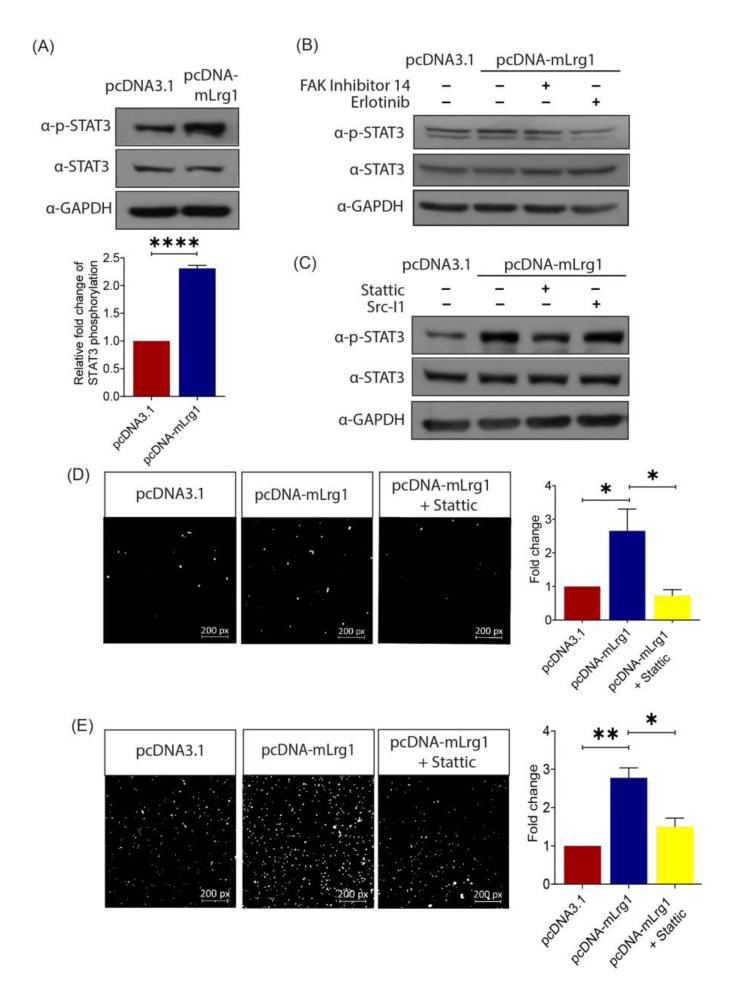
Lrg1-induced activation of the EGFR/STAT3 pathway is required for melanoma cell invasiveness. (**A**) Representative Western blot (left) and densitometry (right) analyses (right) showing the levels of phospho-STAT3 (Tyr705) and total STAT3 in Lrg1 overexpressing B16F10 cells. GAPDH was used as a loading control. (**B**) Representative images of Western blot analysis showing the levels of phospho-STAT3 (Tyr705), STAT3, and GAPDH in Lrg1 overexpressing B16F10 cells subjected to treatment with FAK inhibitor 14 (FAK inhibitor) or erlotinib (EGFR inhibitor). (**C**) Representative images of Western blot analysis showing the levels of phospho-STAT3 (Tyr705) and total STAT3 in Lrg1 overexpressing B16F10 cells subjected to treatment with Src-I1 (Src inhibitor) and stattic (Stat3 inhibitor). GAPDH was used as a loading control. (**D**) Representative images and quantification of migrated Lrg1 overexpressing B16F10 cells subjected to stattic treatment. (**E**) Representative images and quantitative analysis of invaded Lrg1 overexpressing B16F10 cells subjected to stattic treatment. All images are representative. Data are presented as mean ± SEM of three independent experiments. Statistical analyses were performed via two-tailed, unpaired Student’s t-test. * *p* < 0.05; ** *p* < 0.01; **** *p* < 0.0001.

To understand the mechanism of action for Lrg1-mediated melanoma cell activation, Western blot analysis was performed to determine the signalling pathways regulated by Lrg1 in B16F10 cells. Lrg1 was previously reported to regulate EGFR/STAT3 signalling in regenerating corneal epithelium [35]. Our study showed that the level of the phosphorylated form of STAT3 was significantly increased in Lrg1 overexpressing B16F10 cells compared to pcDNA3.1 plasmid transfected control cells, whereas the total STAT3 level remained unchanged (Figure 5A). STAT3 is a transcription factor that is responsible for relaying signals from various activated receptors of cytokines and growth factors, including focal adhesion kinases (FAK) and epidermal growth factor receptors (EGFR) [36]. To identify whether Lrg1-mediated activation of STAT3 occurs through EGFR or FAK, EGFR- and FAK-specific inhibitors erlotinib and FAK inhibitor 14, were used to treat pcDNA3.1 or pcDNA-mLrg1 transfected B16F10 cells. Interestingly, the Lrg1 induced-increase in STAT3 phosphorylation was not affected by the presence of FAK inhibitor 14 but was significantly attenuated in the presence of erlotinib (Figure 5B). Src, a non-receptor protein tyrosine kinase associated with EGFR within lipid rafts [37], was reported to activate STATs directly [38]. To figure out whether Src mediates Lrg1-regulated STAT3 activation, pcDNA3.1 or pcDNA-mLrg1 transfected B16F10 cells were treated with the Src-specific inhibitor Src-I1. Interestingly, Src-I1 did not affect the Lrg1-induced STAT3 phosphorylation (Figure 5C). On the other hand, the STAT3 specific inhibitor significantly inhibited the Lrg1-induced STAT3 phosphorylation (Figure 5C). To further explore whether Lrg1-induced activation of STAT3 signalling is required for Lrg1 regulated melanoma cell invasiveness, Lrg1 overexpressing B16F10 cells with the presence or absence of stattic were subjected to cell migration and invasion assays as described earlier. Lrg1-induced B16F10 cell migration (Figure 5D) and invasion (Figure 5E) were significantly suppressed by stattic. Together, these results suggest that Lrg1 promotes melanoma cell migration and invasion by activating the EGFR/STAT3 signalling pathway in a Src-independent manner.

## 4. Discussion

Malignant melanoma is characterized by its high resistance to chemotherapy and the ability to rapidly metastasize to distant organs. To date, limited treatments are available to control malignant melanoma effectively. A better understanding of melanoma pathogenesis may facilitate the development of new therapeutic modalities. LRG1 is a novel angiogenic factor [7] that was previously associated with a variety of cancers, including endometrial carcinoma, gastric, colorectal, and pancreatic cancers [39,40,41,42]. However, the role of LRG1 in melanoma development and progression has not been established.

Although expressed at low levels in normal melanocytes, we show for the first time that LRG1 is significantly enhanced in malignant melanoma cells of human melanoma tissues. Furthermore, Lrg1 expression levels in the metastatic mouse melanoma cell line B16F10 are significantly higher than those in the parental melanoma cell line B16F0. This observation is consistent with the data from The Human Protein Atlas (https://www.proteinatlas.org/, accessed on 23 April 2021), which show that high LRG1 expression levels are associated with a poor three-year prognosis of patients with melanoma. However, these data do not inform whether the upregulated expression of LRG1 is the cause or consequence of melanoma development and progression.

We used the xenograft tumour model to establish the cause–effect relationship between Lrg1 and melanoma development. Interestingly, the growth of xenografted tumours, the mouse survival rate, and melanoma cell proliferation were not affected in the absence of host *Lrg1*. Considering the large amount of Lrg1 produced by B16F10 cells, we further validated the role of Lrg1 on melanoma cell growth using an in vitro cell-based assay, which again showed no impact of Lrg1 on melanoma cell viability and proliferation. To grow beyond 2 mm^3^, a tumour requires blood vessels to supply oxygen and nutrients [32]. Despite the important role of Lrg1 in ocular angiogenesis, no changes were observed in tumour vessel density in xenograft tumours of wild-type and *Lrg1^-^*^/*-*^ mice. It is possible that Lrg1 derived from implanted B16F10 cells is able to compensate for the loss of Lrg1 in Lrg1-deficient mice, therefore, leaving tumour angiogenesis unaffected. To further elucidate the role of Lrg1 in tumour angiogenesis, future work should compare tumour angiogenesis in Lrg1 knockout mice inoculated with control B16F10 cells, Lrg1 overexpressing B16F10 cells, and B16F10 cells subjected to siRNA-mediated Lrg1 knockdown.

As melanoma is highly metastatic, we next investigated the role of Lrg1 in melanoma dissemination to the lungs. Our finding revealed a significant reduction in tumour burden, total tumour nodule area, and melanoma cell extravasation in the lungs of *Lrg1^-^*^/*-*^ mice as compared to wild-type controls. Successful extravasation depends on the ability of tumour cells to adhere to the endothelial cell and migrate across the endothelium through a process termed transendothelial migration [38,39]. To support in vivo observations, we showed an increased capability B16F10 cells overexpressing Lrg1 to adhere to and migrate across the HPMEC monolayer. Consistent with the in vivo observations, the ability of parental B16F10 cells to adhere to and transmigrate across the HPMEC subjected to siRNA-mediated Lrg1 knockdown was significantly lower, suggesting that both tumour cell and endothelial cell-derived Lrg1 affect melanoma cell extravasation. We further showed that Lrg1 overexpressing B16F10 cells are more migratory and invasive and show increased adhesion to fibronectin. Similar findings were made for glioma cells [43], colorectal cancer cells [22], and thyroid carcinoma cells [44].

STAT-family proteins are latent cytoplasmic transcription factors [45]. Upon phosphorylation, STATs form homodimers and translocate into the cell nucleus to regulate gene expression [34]. Numerous oncogenic signalling pathways converge on STATs proteins, particularly to STAT3 [46,47]. Hyperactivation of STAT3 has been associated with poor prognosis in various malignancies [48,49], including melanoma [34,50,51]. STAT3 exerts its function by promoting tumour metastasis through mediating tumour cell proliferation, migration, and invasion as well as tumour angiogenesis [52]. As such, targeting STAT3 has been considered a promising therapeutic strategy for highly metastatic melanoma. To date, several STAT3 inhibitors have been tested and demonstrated promising results in early-phase clinical trials, but none of them has been approved for melanoma treatment due to adverse side effects and toxicity [53,54]. Therefore, the development of a safer and more effective way to control STAT3 signalling is highly desired. Here, we demonstrated that Lrg1 promotes STAT3 phosphorylation in an EGFR-dependent manner. Furthermore, STAT3 activation is required for the promoting effect of LRG1 on melanoma cell migration and invasion. Since *Lrg1^-^*^/*-*^ mice are viable and show no obvious abnormality, targeting Lrg1 may offer an alternative way to control STAT3-mediated metastasis.

## 5. Conclusions

Overall, our study provided strong evidence of Lrg1’s role in melanoma metastasis and Lrg1 exerting its function through activation of the EGFR/STAT3 signalling pathway. As *Lrg1^-^*^/*-*^ mice are healthy and have a normal life span, unlike mice treated with current STAT3 inhibitors, targeting LRG1 may cause fewer unwanted side effects and offers an alternative strategy to control STAT3-mediated melanoma metastasis.

## Figures and Tables

**Table 1:** Sequences of the forward and reverse primers utilized for gene expression analysis.

Target (Mouse)	Forward Sequence (5’–3’)	Reverse Sequence (5’–3’)
*Lrg1*	TGCACCTCTCGAGCAATCG	AGAGCATTGCGGGTCAGATC
*Endoglin*	CGATAGCAGCACTGGATGAC	AGAATGGTGCCTTTGGGTCT
*Alk1*	CTTGGGGAGCTTCAGAAGGGG	GGTGGCCTCCAGCATCAGAGA
*Alk5*	AAATTGCTCGACGCTGTTCT	GGTACAAGATCATAATAAGGCAACTG
*Gapdh*	ACTGAGGACCAGGTTGTCTCC	CTGTAGCCGTATTCATTGTCATACC

## Supplementary Materials

The following are available online at https://www.mdpi.com/article/10.3390/cancers13133279/s1, Figure S1: Real-time quantitative PCR analysis of Alk5 gene expression, Figure S2: LRG1 promotes A375 cell invasiveness in vitro, Figure S3: Gene silencing efficiency of LRG1 siRNA in HPMEC.
