# Supplementary material for: LRG1 Promotes Metastatic Dissemination of Melanoma through Regulating EGFR/STAT3 Signalling
Source: Cancers (Basel). 2021 Jun 30;13(13):3279. doi: 10.3390/cancers13133279 (PMC8269286; doi:10.3390/cancers13133279)
Supplement: Supplementary file 1 [file cancers-13-03279-s001.zip › cancers-1258902-supplementary.pdf]

Supplementary Materials:

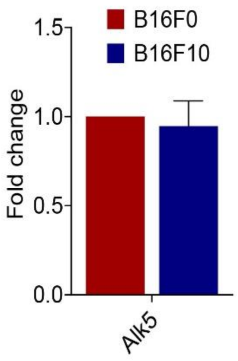

Supplementary Figure S1. Real-time quantitative PCR analysis of Alk5 gene expression. Data are presented as the mean ± S.E.M of three independent experiments. Statistical analysis was performed using two-tailed, unpaired student's t-test.

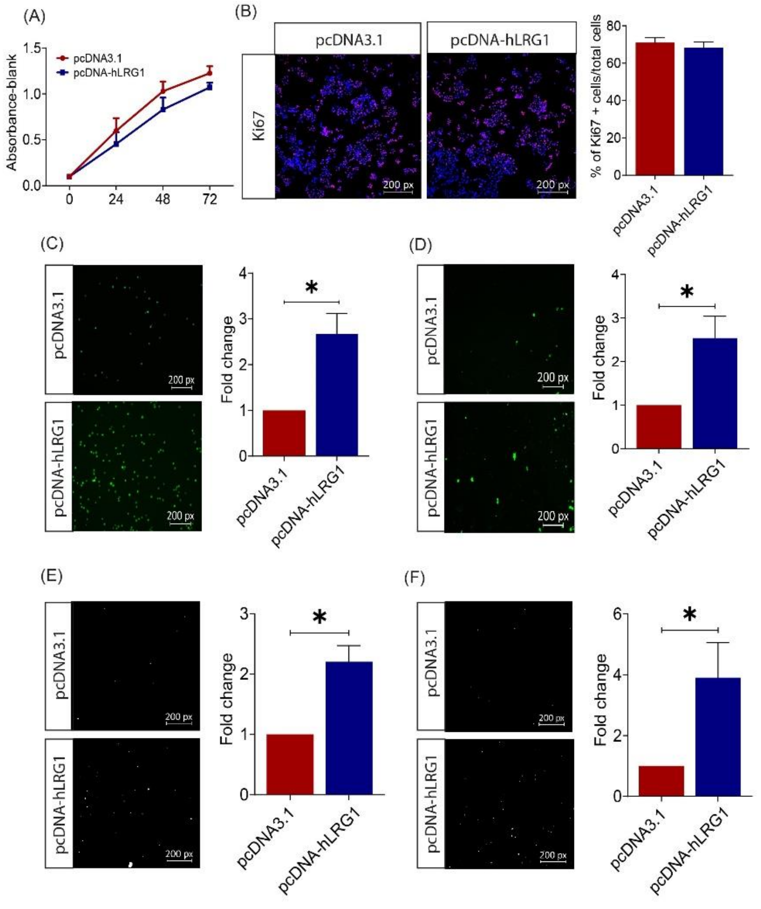

Supplementary Figure S2. LRG1 promotes A375 cell invasiveness in vitro. (A) MTS assay was performed on hLRG1 overexpressing or pcDNA3.1 plasmid transfected A375 cells at 24, 48, and 72 hours post-transfection. (B) Representative images and quantification of Ki67+ cells in hLRG1 overexpressing or pCDNA3.1 plasmid transfected control A375 cells. (C) Representative images and quantitative analysis of migrated LRG1 overexpressing or control A375 cells in Transwell. (D) Representative images and quantitative analysis of the number of CMFDA-positive LRG1 overexpressing or control A375 cells adhered to the HPMEC monolayer. (E) Representative images and quantitative analysis of the number of CMFDA-positive LRG1 overexpressing A375 cells that are migrated through the HPMEC monolayer. (F) Representative images and quantitative analysis of invaded Lrg1 overexpressing and control A375 cells using Matrigel invasion assay. All images are representative.

Data are presented as mean ± S.E.M. of three independent experiments. Statistical analyses were performed using two-tailed, unpaired student's t-test. * $p < 0.05$; ** $p < 0.01$; *** $p < 0.001$.

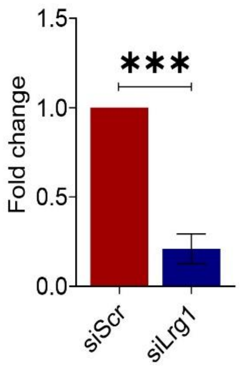

Supplementary Figure S3. Gene silencing efficiency of LRG1 siRNA in HPMEC. The gene expression of LRG1 was detected via real-time quantitative PCR analysis. Data are presented as the mean ± S.E.M of three independent experiments. Statistical analysis was performed using two-tailed, unpaired student's t-test. Statistical analyses were performed using two-tailed, unpaired student's t-test. *** $p < 0.001$.
