# Supplementary figures and images for: LRG1 Promotes Metastatic Dissemination of Melanoma through Regulating EGFR/STAT3 Signalling
Source: Cancers (Basel). 2021 Jun 30;13(13):3279. doi: 10.3390/cancers13133279 (PMC8269286; doi:10.3390/cancers13133279)

Figure 5A

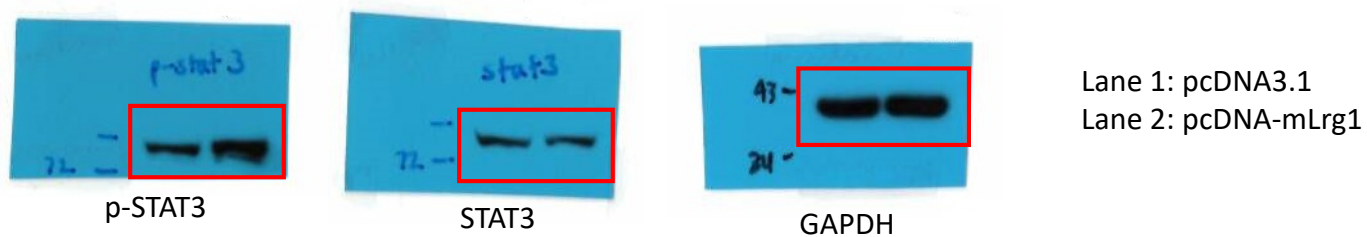

Figure 5B

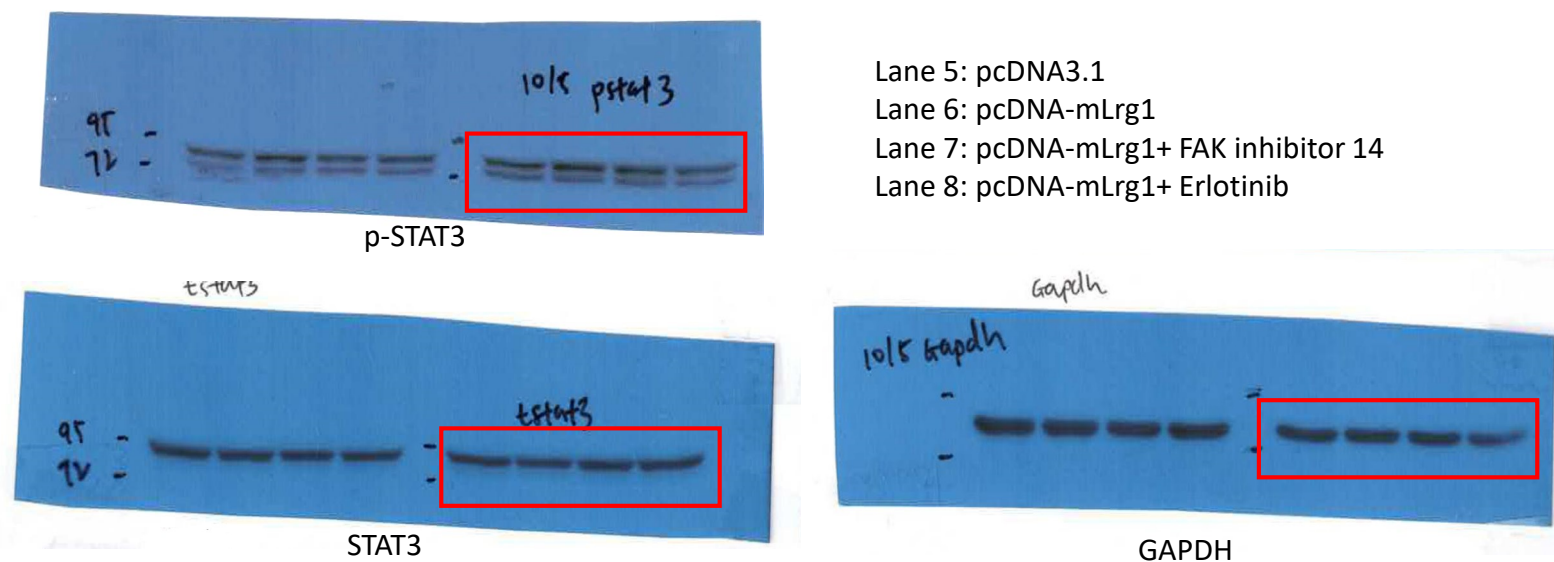

Figure 5C

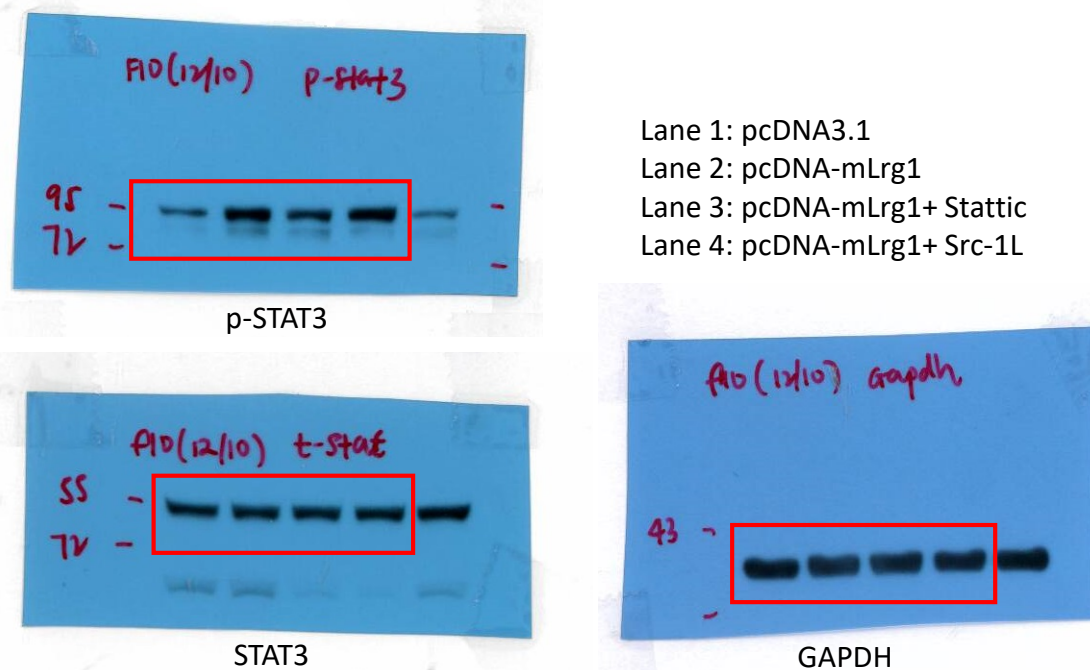

Supplement: Supplementary file 1 [file cancers-13-03279-s001.zip › Western blots.pdf]
